# Quality indicators for systemic anticancer therapy services: a systematic review of metrics used to compare quality across healthcare facilities

**DOI:** 10.1016/j.ejca.2023.113389

**Published:** 2023-12

**Authors:** Kari Leung, Megan McLeod, Julie Torode, André Ilbawi, Jade Chakowa, Brian Bourbeau, Manju Sengar, Christopher M. Booth, Julie R. Gralow, Richard Sullivan, Ajay Aggarwal

**Affiliations:** a Department of Oncology, Guy’s & St Thomas’ NHS Foundation Trust, London, UK; b Vanderbilt University School of Medicine, Nashville, Tennessee, USA; c Institute of Cancer Policy, King’s College London, London, UK; d World Health Organization, Geneva, Switzerland; e City Cancer Challenge, Geneva, Switzerland; f American Society of Clinical Oncology, Alexandria, Virginia, USA; g Department of Medical Oncology, Tata Memorial Centre, Mumbai, India; h Division of Cancer Care and Epidemiology, Departments of Oncology and Public Health, Queen's Cancer Research Institute, Queen's University, Kingston, Ontario, Canada; i Department of Health Services Research and Policy, London School of Hygiene and Tropical Medicine, London, UK

**Keywords:** Systemic anticancer therapy, Quality improvement, Quality indicator, Quality of care

## Abstract

**Purpose:**

The number of systemic anticancer therapy (SACT) regimens has expanded rapidly over the last decade. There is a need to ensure quality of SACT delivery across cancer services and systems in different resource settings to reduce morbidity, mortality, and detrimental economic impact at individual and systems level. Existing literature on SACT focuses on treatment efficacy with few studies on quality or how SACT is delivered within routine care in comparison to radiation and surgical oncology.

**Methods:**

Systematic review was conducted following PRISMA guidelines. EMBASE and MEDLINE were searched and handsearching was undertaken to identify literature on existing quality indicators (QIs) that detect meaningful variations in the quality of SACT delivery across different healthcare facilities, regions, or countries. Data extraction was undertaken by two independent reviewers.

**Results:**

This review identified 63 distinct QIs from 15 papers. The majority were process QIs (n = 55, 87.3%) relating to appropriateness of treatment and guideline adherence (n = 28, 44.4%). There were few outcome QIs (n = 7, 11.1%) and only one structural QI (n = 1, 1.6%). Included studies solely focused on breast, colorectal, lung, and skin cancer. All but one studies were conducted in high-income countries.

**Conclusions:**

The results of this review highlight a significant lack of research on SACT QIs particularly those appropriate for resource-constrained settings in low- and middle-income countries. This review should form the basis for future work in transforming performance measurement of SACT provision, through context-specific QI SACT development, validation, and implementation.

## Highlights

• Sixty-three systemic therapy quality indicators were identified from 15 papers.
• Majority of quality indicators focused on processes of care delivery.
• Included studies focused on breast, colorectal, lung, and skin cancer.
• There was limited research on quality indicators applicable to LMICs.

## 1 Introduction

The global cancer burden, estimated at 19.3 million new cases worldwide in 2020, is projected to rise to 27.5 million by 2040 [1], [2]. The majority of these patients will require systemic anticancer therapies (SACT) in both curative and palliative settings [3]. The number and complexity of SACT regimens have expanded rapidly over the last decade with the introduction of immunotherapy, small molecule targeted cancer medicines, and cell therapies such as chimeric antigen receptor-T cell therapies [4], [5], [6], [7]. But with increasingly complicated treatment regimens comes the challenge of ensuring the quality of SACT delivery across very different cancer services and systems around the world.

Unsafe and inappropriate use of SACT is a cause of avoidable treatment-related mortality and morbidity [8]. Appropriate and safe delivery of SACT involves consideration of many factors such as dose, interval, indication, supportive medicines and services, route of administration, and timeliness of treatment. Variation in the quality of SACT service delivery directly impacts immediate treatment outcomes, survival, and the quality of life of patients. The need to standardise and monitor quality through appropriate context-specific indicators is critical to promote best practice in the delivery of SACT services, as exemplified by a recent partnership between the American Society of Clinical Oncology (ASCO) and the World Health Organization (WHO) [9]. The collaboration aims to measure and improve the quality of cancer care internationally, particularly in low- and middle-income countries (LMICs).

Quality indicators (QIs) are measurement tools to evaluate the quality of care. QIs are used across health services to measure compliance with evidence-based standards set by national and international organisations and can be applied on a regional, national, or international level [10]. They are also essential to organisations and institutions internally to ensure consistency, to identify gaps, and to prioritise areas for improvement. Quality improvement is the framework for systematically improving care through standardised processes and structure to reduce variation, achieve predictable results, and improve outcomes for patients, healthcare systems, and organisations [11]. QIs form the basis for healthcare performance assessment and quality improvement through benchmarking of best practice, encouraging internal provider-level process review and audit, as well as stimulating market-based incentive for quality improvement through patient choice and hospital competition [12].

Most QIs in healthcare are conceptually derived from the Donabedian quality framework, whereby QIs fall into three categories: structure, process, and outcome [10]. Structural QIs refer to the setting in which care is delivered: resources, such as the quantity and availability of buildings and equipment; human resources, such as staffing levels and qualifications; and timeliness. Process QIs refer to the delivery and receipt of care: adherence to guidelines; appropriateness of treatment; and multidisciplinary coordination of care. Outcome QIs refer to the resultant effects of receiving care on the patient and general population: mortality; safety and adverse effects; patient-reported outcome measures (PROMs). Further models have also been derived from this basic framework; for example, the 12 domain model of quality in cancer care with additional domains covering technical aspects, innovation, and value [13].

In cancer, surgical oncology has led the way in quality improvement studies [14], [15], [16], [17], [18], [19], [20], closely followed by radiation oncology [21], [22]. Conversely, research into QIs for SACT or medical oncology has been relatively limited, with little literature that seeks to understand the delivery of SACT within routine care or the quality of such delivery [23], [24].

This systematic review seeks to address a gap in the knowledge by establishing what QIs are being used to assess the provision of SACT to compare different healthcare facilities, regions, or countries, with an aim to establish practical QIs that can detect meaningful variations between healthcare facilities. This review also explores the purpose of QI reporting in these studies and whether any results were fed back to the respective organisations or to support quality improvement activities.

## 2 Methods

### 2.1 Literature search strategy

This study used the Population, Intervention, Comparison, and Outcomes framework to create the search strategy. A literature search was carried out between 1st and 5th November 2022, searching EMBASE and MEDLINE to identify relevant literature pertaining to the use of QIs in SACT delivery. Records were extracted between 1st January 2000 and 31st October 2022 in order to reflect contemporary literature and were reported following the Preferred Reporting Items for Systematic Reviews and Meta-Analyses guidelines [25]. The database searches were supplemented with handsearching of studies eligible for inclusion in the review by K.L. and M.M. A detailed description of the database search strategy can be found in Appendix A. The grey literature was not formally searched or included in this review.

The inclusion and exclusion criteria were developed alongside expert consultation with an oncologist and surgeon, both senior reviewers (A.A. and R.S.).

### 2.2 Inclusion criteria

Original studies published in English that utilised QIs of SACT in adult patients to compare quality of care between hospitals, regions, or countries, or against a national average or predetermined standard as indicated by national guidelines.

### 2.3 Exclusion criteria

Studies that developed, proposed, or validated QIs (but did not implement them), as well as studies that examined associations and causal factors for SACT QI measurements (e.g., patient demographics). Case reports, abstracts, professional organisation guidelines, conference proceedings, and opinion articles were excluded.

Title and abstract screening were undertaken by K.L. and M.M. and a senior reviewer (A.A.) before proceeding onto full-text review where studies were evaluated against the inclusion criteria. Discrepancies and uncertainties were resolved with the involvement of another expert reviewer (R.S.).

### 2.4 Data extraction and data synthesis

A standardised form was generated to extract data in a systematic manner; study characteristics and defined QIs were recorded. Study characteristics extracted were year of publication, study country, source of study data, period of study, number of hospitals evaluated, number of patients, tumour type(s), and staging. Data extraction was undertaken by the primary reviewers (K.L. and M.M.); senior reviewers (A.A. and R.S.) were consulted to resolve discrepancies. QIs extracted from studies were classified using Donabedian domain (structure, process, and outcome) and were grouped into themes that emerged from final dataset of QIs. Descriptive statistics were used to summarise study findings. Covidence® software was utilised to remove duplicates, screen titles, and abstracts; data were extracted into Microsoft® Excel.

## 3 Results

### 3.1 Description of studies

**Fig. 1:**
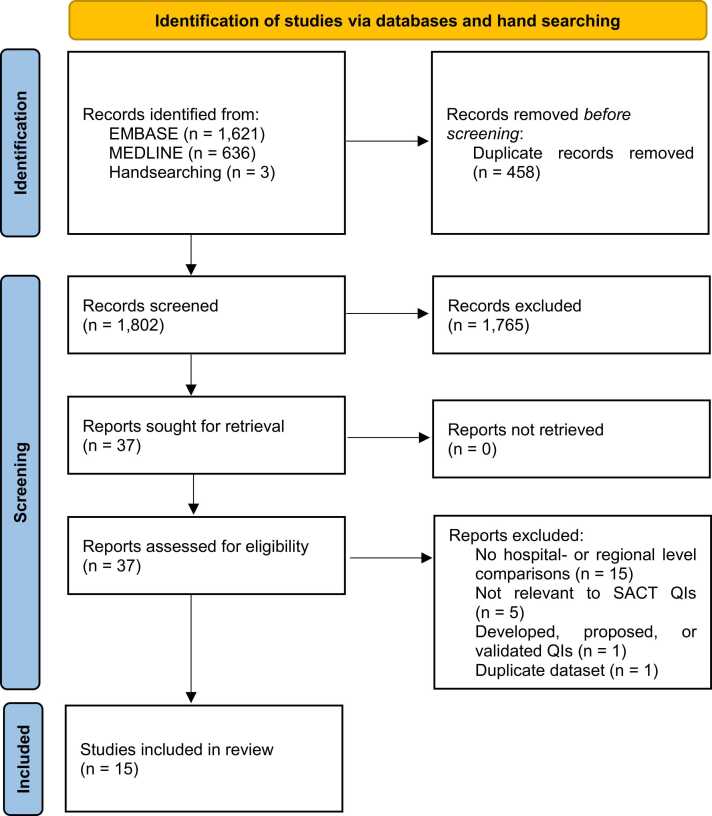
Preferred Reporting Items for Systematic Reviews and Meta-Analyses diagram. QI, quality indicator; SACT, systemic anticancer therapy.

The literature searches yielded 1799 unique articles. Of the 34 full-text articles for reviewed, 13 studies were suitable for inclusion in this review (Fig. 1). Twenty-one studies were excluded primarily due to a lack of hospital- or regional-level comparisons or were not specific to SACT care. Handsearching of the references of included studies yielded an additional three papers, of which two were eligible for inclusion. Thus, 15 studies were included in this systematic review.

Studies were identified from the United States of America (USA) (n = 3, 20.0%) [26], [27], [28]; Netherlands (n = 3, 20.0%) [29], [30], [31]; Canada (n = 2, 13.3%) [32], [33]; United Kingdom (n = 2, 13.3%) [34], [35]; Germany, Italy, Austria, and Switzerland combined (n = 2, 13.3%) [36], [37]; Japan (n = 1, 6.7%) [38]; Portugal (n = 1, 6.7%) [39]; and South Africa (n = 1, 6.7%) [40]. Only one study was conducted in a middle-income country, South Africa [40]. No studies were conducted in low-income country settings. All studies compared results between different healthcare facilities [26], [27], [36], [37], [38], [39], [40], [28], [29], [30], [31], [32], [33], [34], [35].

The number of patients included in each study varied widely from 595 to 253,182 patients (IQR 6018–120,038), with a total of 1,036,310 patients overall. The number of healthcare facilities examined ranged from 2 to 1324 (IQR 10–235). The studies examined four tumour groups: breast (n = 11, 73.3%) [26], [28], [40], [30], [32], [33], [35], [36], [37], [38], [39], lung (n = 4, 26.7%) [27], [28], [31], [35], colorectal (n = 2, 13.3%) [28], [34], and skin (n = 1, 6.7%) [29]. Nine out of 15 papers (60.0%) solely focused on breast cancer patients [26], [30], [32], [33], [36], [37], [38], [39], [40], and one paper investigated patients from three tumour groups [28]. Seven studies (46.7%) included all tumour stages (stages I to IV) [26], [28], [29], [30], [35], [37], [38], and the remainder selected discrete tumour stages [27], [31], [32], [33], [36], [39], [40], [41].

### 3.2 SACT QIs

Sixty-three distinct QIs were extracted from the 15 studies, falling into eight broad themes: 1) appropriateness of care and guideline adherence, 2) treatment planning and immunohistochemistry (IHC) testing, 3) treatment time intervals, 4) adverse events and side-effects, 5) multidisciplinary and coordinated care, 6) mortality, 7) supportive medicines, and 8) electronic prescribing systems. Appropriateness of care and guideline adherence constituted the largest group (n = 28, 44.4%) [26], [27], [28], [29], [30], [33], [36], [37], [38], [39], followed by treatment planning and IHC testing (n = 14, 22.2%) [26], [30], [33], [37], [38], and treatment time intervals (n = 8, 12.7%) [26], [30], [33], [40]. The number of QIs utilised in the studies reviewed ranged from one to 17 [26], with nine out of 15 (60.0%) reporting a single QI related to SACT [27], [28], [29], [31], [32], [34], [35], [36], [39]. A breakdown of the major themes and QIs extracted can be found in Table 2, and QIs classified by study is available in Appendix B.

Process QIs were the most prevalent with 55 out of 63 QIs (87.3%) [26], [27], [40], [28], [29], [30], [33], [36], [37], [38], [39] relating to adherence to national and international guidelines for evidence-based practice published by professional organisations, such as ASCO and the European Society of Medical Oncology (ESMO). This is mostly quantified as proportion of patients that have guideline-adherent treatment. Twenty-five QIs (39.7%) related to IHC testing, florescence in situ hybridisation (FISH) testing, or appropriateness of treatment linked to human epidermal growth factor receptor 2 (HER-2), oestrogen receptor (ER), and progesterone (PR) status in breast cancer patients [26], [30], [33], [36], [37], [38], [40].

Seven outcome QIs were identified (11.1%) which related to death following SACT, prevalence of side-effects and adverse events, and hospitalisation and emergency department (ED) visits [31], [32], [33], [34], [35]. Only one outcome diagnosis, serious febrile neutropenia, was named in a QI. Two QIs investigated rates or episodes of ED visits and hospitalisations [32], [33]. One QI sought to determine hospital-level toxicity rates during SACT treatment by quantifying cases where severe acute toxicities required an overnight hospital admission [34].

Only one structural indicator (1.6%) was identified from the studies reviewed [33]; this related to the availability of electronic chemotherapy prescribing systems.

### 3.3 Purpose of SACT QI reporting and feedback

Seven studies sought to determine adherence to QIs [26], [27], [30], [32], [38], [39], [40]; three studies aimed to demonstrate evidence of variation in practice [31], [34], [37] (i.e., to determine healthcare facilities that are outliers); two studies investigated reasons for non-conformity to QIs [28], [36]; two studies used administrative data to define achievable targets for QIs and national benchmark rates [33], [35]; and one study aimed to show that claims data could be used to benchmark cancer care and assess clinical practice variation [29].

Reasons for comparative performance assessment of healthcare facilities were primarily for academic purposes (n = 12, 80%) [26], [27], [28], [29], [31], [32], [33], [35], [36], [38], [39], [40], with three papers being conducted as part of national audits (20.0%) [30], [34], [37]. Five out of 15 studies (33.3%) performed risk adjustment in their statistical analysis [27], [31], [34], [35], [40]. Two studies (13.3%) reported their findings back to the assessed healthcare facilities [26], [30], two (13.3%) studies stated their findings were publicly reported [34], [35], and one (6.7%) study reported findings back to healthcare facilities involved and published results anonymously to the public [37]. Further details of study characteristics can be found in Table 1.

**Table 1:** Characteristics of included studies.

Study; year	Country	Number of healthcare facilities evaluated (No. of patients)	Tumour group (s); stage	Units of comparison	Comparator	Purpose	Reason for reporting	Feedback	Risk adj.
Burgers; 2018 [31]	Netherlands	77 (n = 26,277)	Lung; III to IV	Other facilities; national level	Literature-defined (death rate)	Evidence of variation in practice	Academic	Yes	No
Khorfan; 2021 [27]	USA	1324 (n = 253,182)	Lung; I to II	Other facilities	Guidelines (National)	Measure adherence to QIs	Academic	Yes	No
Van Egmond; 2021 [29]	Netherlands	124 (n = 221,880)	Skin; all stages	Other facilities	N/A	Proving data source could be used to benchmark skin cancer care and assess clinical practice variation	Academic	No	No
O'Neil; 2019 [40]	South Africa	5 (n = 1736)	Breast; I to III	Other facilities	Guidelines (National)	Measure adherence to QIs	Academic	Yes	No
Ellis; 2020 [28]	USA	1281 (n = 183,148)	Breast, colorectal, lung; all stages	Other facilities	Guidelines (National)	Evidence of variation in practice; reasons for non-conformity to guidelines	Academic	No	No
van Bommel; 2017 [30]	Netherlands	92 (n = 56,927)	Breast; all stages	Other facilities	Guidelines (National)	Measure adherence to QIs	National audit	No	Yes
Mukai; 2015 [38]	Japan	224 (n = 15,227)	Breast; all stages	Other facilities	Guidelines (National)	Measure adherence to QIs	Academic	No	No
Wu; 2021 [32]	Canada	2 (n = 6336)	Breast; I to III	Other facilities; regional level	N/A	Measure adherence to QIs	Academic	No	No
Inwald; 2019 [36]	Germany, Italy, Austria, Switzerland	274 (n = 5700)	Breast; I to III	Other facilities	Guidelines (National)	Reasons for non-conformity to guidelines	Academic	No	No
Powis; 2017 [33]	Canada	84 (n = 28,303)	Breast; I to III	Other facilities	N/A	Using administrative data to define achievable targets for QIs	Academic	No	No
Gray; 2011 [26]	USA	11 (n = 622)	Breast; all stages	Other facilities	Guidelines (National)	Measure adherence to QIs	Academic	No	Yes
Ferreira; 2016 [39]	Portugal	4 (n = 595)	Breast; I	Other facilities	Literature-defined (USA)	Measure adherence to a certain outcome	Academic	No	No
Boyle; 2022 [34]	United Kingdom	106 (n = 8173)	Colorectal; III to IV	Other facilities; national mean	Literature-defined (national mean)	Evidence of variation in practice, identification of outliers	National audit	Yes	Yes
Wallington; 2016 [35]	United Kingdom	147 (n = 32,862)	Breast, lung; all stages	Other facilities	N/A	Identification of outliers; establish national 30-day mortality benchmark	Academic	Yes	Yes
Kowalski; 2014 [37]	Germany, Italy, Austria, Switzerland	268 (n = 195,342)	Breast; all stages	Other facilities	Guidelines (National)	Evidence of variation in practice, identification of outliers	National audit	Yes	No

Adj, adjusted; QI, quality indicator; USA, United States of America.

**Table 2:** Quality indicators.

Donabedian domain/Theme	Quality indicators
Process	
Appropriateness of care and guideline adherence	Percentage of skin cancer cases treated with 5-fluorouracil or imiquimod
	Percentage of hormonally-sensitive patients who received adjuvant hormone therapy within 1 year of surgery
	Percentage of patients who received chemotherapy where the chemotherapy regimen was from the provided list
	Percentage of non-metastatic patients whose planned dose of chemotherapy was documented, where the patient’s planned dose of chemotherapy, dose per cycle, and number of cycles fell within a range that is consistent with published regimens
	Percentage of non-metastatic patients with ER- or PR-positive breast cancer where the physician discussed, recommended, or referred treatment with tamoxifen or aromatase inhibitors within 1 year of diagnosis
	Percentage of patients recommended endocrine therapy in cases of steroid receptor diagnostic finding
	Percentage of patients who initiated multiagent chemotherapy within 120 days of surgery
	Percentage of patients with HER-2 negative breast cancer who received trastuzumab within 12 months of diagnosis
	Percentage of patients with stage II or higher breast cancer recommended SACT, or reason is documented if not recommended
	Percentage of patients with positive HER-2 status where the physician discussed, recommended, or offered treatment with trastuzumab
	Percentage of patients with stage I, ER- or PR-negative disease and tumour size >1 cm where the physician discussed, recommended, or referred for chemotherapy
	Percentage of patients with stage II or III disease where the physician discussed, recommended, or referred for chemotherapy
	Percentage of stage IV patients with ER- or PR-positive breast cancer where the physician discussed, recommended, or referred treatment with tamoxifen, fulvestrant, or aromatase inhibitors within 1 year of diagnosis
	Proportion of patients with invasive M0 breast cancer who received neo-adjuvant or postoperative chemotherapy
	Proportion of invasive breast cancer cases, ER- or PR-positive, and tumour diameter ≥1 cm that received postoperative hormone therapy
	Proportion of adjuvant chemotherapy breast cancer cases that received a regimen including anthracyclines, taxanes, or CMF
	Proportion of postoperative invasive breast cancer cases which received therapy adherent to St. Gallen consensus recommendations
	Proportion of patients with early HER-2 positive breast cancer where rationale for giving or not giving trastuzumab is documented
	Rates of failure to administer recommended chemotherapy (hospital level)
Treatment planning and IHC	Percentage of patients who received chemotherapy where the patient's body-surface area was documented
	Percentage of patients who received chemotherapy where patient’s planned dose of chemotherapy was documented in their notes
	Percentage of patients who received chemotherapy where there was a flowsheet with chemotherapy notes and blood counts
	Percentage of patients who received chemotherapy where there is evidence of signed or documented consent to treatment
	Percentage of patients with pretherapeutic histological confirmation
	Proportion of patients with primary invasive breast cancer and availability of ER, HER-2, or PR status
	Proportion of patients with invasive breast cancer with standard pathology report including information about ER percentage, PR percentage, HER-2 status, malignancy grade, tumour size, resection margin, and number of positive lymph nodes
Treatment time intervals	Percentage of non-metastatic patients who received adjuvant chemotherapy that started within 4 months of diagnosis
	Percentage of non-metastatic patients who received adjuvant chemotherapy that started within 8 weeks of completing surgical therapy
	Percentage of patients who initiated chemotherapy within 60 days of surgery
	Proportion of patients receiving SACT with transit time ≤5 weeks between end of radiotherapy or final operation and start of SACT
	Proportion of patients receiving neo-adjuvant chemotherapy with transit time ≤5 weeks between diagnosis and start of chemotherapy
	Proportion of women with AJCC stage I–III disease, tumour size >1 cm, and ER- or PR-positive status who receive Tamoxifen or an aromatase inhibitor within 365 days from diagnosis
	Proportion of women with AJCC stage II–III disease, ER- and PR-negative status who receive SACT within 120 days from diagnosis
Multidisciplinary and coordinated care	Percentage of non-metastatic patients who received neo-adjuvant chemotherapy where they received definitive surgery after SACT
	Percentage of patients on hormone therapy with >80% of eligible days covered with prescription
	Percentage of patients with at least one consultation with a provider that prescribes chemotherapy within 120 days of surgery
Supportive medicines	Percentage of patients who filled a prescription for appropriate antiemetics with their cycle of chemotherapy
	Percentage of patients who had an emergency department visit and hospitalisation episode with at least one prescription filled for GCSF
Outcome	
Adverse events and side-effects	Percentage of patients who had an emergency department visit or hospitalisation episode within 30 days of SACT
	Rates of emergency department visits and hospitalisations in the 180 days following chemotherapy initiation
	Percentage of patients that received SACT who experienced serious treatment-related neutropenia within 30 days of SACT
	Rates of hospital-level severe acute toxicity (i.e., requiring an overnight hospital admission) for patients receiving chemotherapy
Mortality	30-day mortality rates for breast and lung cancer patients receiving chemotherapy
	Percentage of patients who died within 60 days of adjuvant chemotherapy
	Proportion of patients with 30-day mortality after the start of systemic treatment for lung cancer
Structure	
Electronic prescribing system	Percentage of patients whose first cycle of chemotherapy was ordered using the computerised provider order entry system


AJCC, American Joint Committee on Cancer; CMF, cyclophosphamide, methotrexate, and fluorouracil; ER, oestrogen receptor; GCSF, granulocyte colony-stimulating factor; HER-2, human epidermal growth factor receptor 2; IHC, immunohistochemistry; PR, progesterone receptor; SACT, systemic anticancer therapy.

## 4 Discussion

This systematic review of contemporary literature on QIs for SACT identified 63 unique metrics from 15 studies in the last 20 years across only four site-specific cancers: breast, lung, colorectal, and skin. All but one of these studies were conducted in high-income settings. Using the Donabedian domains framework, the majority of QIs identified (n = 55, 87.3%) were classified as process QIs, seven were classified as outcome QIs, and only one was classified as a structural QI. The most common domains identified from extracted QIs were appropriateness of care and guideline adherence, followed by treatment planning and IHC testing, and treatment time intervals.

While only four tumour groups were represented, these broadly aligned to the most common cancers globally [42]. The omission of haemato-oncology, which uses different regimens to solid cancers as well as methods of administration such as intrathecal administration of SACT, means there is a lack of QIs that capture the high potential for harm associated with some SACT agents. Moreover, many other elements of care that are tumour-group-specific are absent from the QIs extracted; for example, BRCA1 and BRCA2 genetic testing to guide treatment decisions in ovarian cancer as per the Society of Gynaecologic Oncology [43].

### 4.1 Process QIs

The majority of QIs (n = 55, 87.3%) identified were process metrics, specifically appropriateness of care and adherence to local, national, or international guidelines. This finding is consistent with existing literature on QIs in other domains of cancer care, such as radiotherapy [13]. In high-income countries (HICs), data for these QIs are usually routinely available and collected within patient notes, SACT databases, or accessible through insurance claims.

Guidelines are formulated and published on national and international scales through professional associations; for example, ASCO (USA), ESMO (Europe), and aim to minimise inappropriate treatment variation and optimise patient outcomes. Guidelines may consider cost-effectiveness and other factors beyond clinical outcomes. Therefore, guideline adherence QIs are good examples of performance metrics that meaningfully indicate high-quality care. High adherence to guidelines has been shown to be associated with better survival outcomes; a systematic review and meta-analysis on breast cancer guideline adherence concluded with moderate certainty that this was associated with improved survival [44].

Twenty-five process QIs referred to molecular profiling (e.g., ER/PR and HER-2 status) in breast cancer prior to treatment delivery and was found in seven of the 11 studies that included breast cancer in their tumour groups of focus [26], [30], [33], [36], [37], [38], [40]. High adherence to molecular profiling in settings using regimens with targeted SACT is suggestive of higher-quality care, as clinicians are able to prescribe the most appropriate treatments based on the full clinical picture [45].

Low adherence rates are indicative of little or no molecular testing being undertaken, suggesting either: 1) more advanced SACT is not available (due to lack of affordability or accessibility), or 2) targeted therapies are being utilised without molecular guidance. In both cases, inappropriate treatment may be given, and in the latter scenario represents a waste of resources (either from the perspective of the healthcare system, or the patient if paying out-of-pocket), unnecessary delays to appropriate treatment, or exposure to unnecessary treatments which may result in adverse side-effects. The large number of QIs identified that focused on IHC testing highlights the significance and role of molecular genetics for SACT, particularly in the era of targeted therapies and immuno-oncology.

The relationship between process QIs and improved patient outcomes has been the subject of debate [46]. In theory, a meaningful process QI should be a surrogate for improved patient outcomes. The strength of the association between a process QI and patient outcomes is described as the ‘contributional validity’ of the process QI [47] and can often be ambiguous without direct clinical evidence from randomised controlled trials in clinical situations. However, these are often unfeasible to undertake for ethical or logistical purposes. Nonetheless, process QIs are typically preferred due to ease of data collection. Powis et al. [33] were able to use process QIs to identify outlying hospitals (defined as performing worse than the 95% confidence interval), allowing quality improvements to be targeted at a hospital level.

### 4.2 Outcome QIs

Outcome QIs are the most direct measurements for quality of care. Seven outcome QIs were identified from the studies reviewed [31], [32], [33], [34], [35] of which three examined rates of mortality within 30 and 60 days of treatment at the hospital level [31], [33], [35]. Mortality data are routinely collected as part of national registries and can be a useful QI as high-quality care should correlate with lower mortality rates. The studies reviewed had mixed success in gathering enough data on mortality to be sufficiently powered to detect outliers; Burgers et al. [31] required 6 years of data to detect three outliers, indicating a lack of sensitivity, whereas Wallington et al. [35] were able to identify several outliers with a single-year dataset. Burgers et al. [31] suggest that linking death with reasons for death would improve sensitivity. However, this should not be necessary for a sufficiently powered study and would be performed as part of a follow-up audit within an individual institution to understand reasons for an outlier’s poor performance. While mortality is a useful QI, it should not be considered in isolation for the quality of SACT but alongside other dimensions.

Three QIs measured severe toxicity resulting in emergency hospitalisations related to SACT [32], [33], [34]. Neutropenic sepsis, a potentially fatal complication of SACT that accounts for 2–21% of SACT mortality rates, was highlighted by one QI [48]. This is linked to a process QI on the provision of granulocyte colony-stimulating factor for secondary prophylaxis of neutropenic sepsis, emphasising the significance of patient education, early identification, swift management, and treatment to minimise avoidable deaths.

Serious SACT toxicity creates patient distress, significant burdens for healthcare facilities with additional costs, intensive use of resources, and contributes to mortality. Boyle et al. [34] developed and applied a QI that captures any severe acute toxicity rates (defined as patients requiring hospital admission due to acute toxicity, at least grade 3 according to the Common Terminology Criteria for Adverse Events) for all colorectal patients receiving SACT within the English NHS. The QI provides granularity between specific toxicities, allowing detailed feedback provision to healthcare facilities and leading to continuous, targeted local quality improvement. This QI could be applied across other tumour groups, countries, and regions where administrative datasets are available.

A common strategy to utilise outcome QIs is to look at performance outliers, typically hospitals with outcomes at two or three standard deviations from the mean outcomes across all hospitals. This strategy requires the outcome QI to be sensitive, risk-adjusted, and sufficiently powered. Risk-adjustment accounts for systematic variation in patient demographics and disease profile between healthcare facilities, ensuring that QIs are a fair assessment of quality. If outcome variation is detected, then further investigation or internal audits should be undertaken to determine underlying causes. Examples of potential areas within the SACT care pathway that may result in variation in care include access (e.g., timely receipt of treatment, appropriateness of treatment), delivery of care (e.g., appropriate dosing and scheduling of treatment, competently trained staff to deliver SACT), and safety (e.g., access to acute oncology services, available supportive protocols for managing toxicities) [34].

The use of PROMs was notably absent from extracted QIs. This may be due to a lack of data available, challenges in collecting data, or challenges in linking results to patient records. QIs reliant on electronic health records and linked registration data can be subject to data quality issues such as accuracy and completeness, which may affect their utility, and data capture and completeness should also be considered within future QIs as a way of raising the standard. A systematic review conducted by Nguyen et al. [49] examining barriers associated with employing PROMs in oncology reported challenges at the patient level (e.g., time required and difficulty in using electronic devices to complete PROMs), professional level (e.g., lack of knowledge and time to meaningfully interpret data, inability to act upon findings), and service level (e.g., difficulties integrating PROMs into existing clinical workflow, inadequate technology infrastructure for routine data collection).

Future work should seek to understand how PROMs data can be routinely collected, developed, integrated, and validated as QIs in cancer care. While concerns have been relayed about having insufficient power to detect significant differences in outcome, there are examples of successful application of PROMs in performance assessment. A study demonstrated that it was feasible to develop outcome indicators, including PROMs for radiotherapy, through the National Prostate Cancer Audit conducted in England and Wales National Health Service (NHS) [21]. PROMs such as bowel and sexual function of patients were linked to administrative datasets, which allowed for relevant risk-adjustment.

### 4.3 Structural QIs

Only one structural QI was identified which related to availability and access to electronic prescribing systems [33]. SACT has high potential for harm, and prescribing is complex due to variable dosing typically linked to body-surface area, making medication errors more likely to occur. A systematic review by Ammenwerth et al. [50] reported a relative risk reduction due to electronic prescribing across 25 studies of 13–99% in medication errors and 35–98% in adverse drug events. Medication errors can also cause significant patient harm and financial cost: one study estimated that medication errors in the UK are the cause of 1700 avoidable deaths and cost the NHS £98 million every year [51]. While the availability of electronic prescribing likely correlates with improved outcomes through more accurate prescribing, a more direct metric is the number of medication errors identified. An improved QI might look at medication errors directly, for which the introduction of electronic prescribing can be considered an initiative to help reduce medication error rates.

### 4.4 Future avenues for research

The findings of this review highlight a major deficit in SACT QI studies conducted in low- and middle-income settings, with only one study carried out in a middle-income country (South Africa) [40]. For this reason, the findings of this review are difficult to extrapolate to systems outside high-income settings and offer little insight into how we can determine the quality of SACT in resource-constrained environments. There are many challenges to utilising the QIs identified in this review in LMICs. For example, determining the proportion of patients that are appropriately treated with trastuzumab in HER2-positive breast cancer is not possible if HER-2 testing is not routinely carried out due to resource and workforce limitations. It is imperative to tailor cancer treatments and strategies to the level of available resources by country, but there is little guidance on how to do this effectively. The National Comprehensive Cancer Network® Framework for Resource Stratification [52] defines appropriate treatment pathways and options for the best possible outcomes in resource-constrained settings and may be a suitable mechanism to develop QIs appropriate for LMICs. The lack of SACT QI research germane to LMICs also means the exclusion of problems that affect these countries, such as supply chain issues, quality of medicines, or counterfeit medicines. According to the WHO, it is estimated that 1 in 10 medicines is substandard or falsified in LMICs, which can seriously undermine efforts to deliver high-quality cancer care [53].

Overall, this review highlights a significant lack of research on SACT QIs given the small number of studies we identified. Additionally, studies which focus on tumour groups not represented in this study are needed to provide a more complete view of the landscape of SACT delivery, although many of the SACT QI indicators can be considered to be tumour agnostic or translatable to other tumour types.

A broader review with a less restrictive search strategy could examine the use of QIs to determine quality of care within healthcare facilities over time for instance, thus including studies which are single-site audits. Moreover, this review did not include searches for grey literature which would include works by governmental and professional organisations. Such guidelines are operational documents rather than *de novo* research on QI, and thus excluded from this review. Examples include ASCO (USA) [54] and European Cancer Centre (Germany) [55].

ASCO’s Quality Oncology Practice Initiative is an example of a quality improvement programme currently active in 38 countries [54]. The programme will utilise data, expert input, and WHO tools to develop evidence-based QIs to assess quality of care of healthcare facilities. The pilot programme is currently underway and initial findings are expected to be published in 2024. Moreover, ASCO intends to expand its utilised QIs at additional LMIC sites of care in partnership with WHO to achieve health-related targets of the U.N. Sustainable Development Goals and WHO Global Action Plan on Non-Communicable Diseases [9].

Risk of bias was not undertaken for this review, and studies were included regardless of methodological strength because we were concentrating on the type of indicators routinely used rather than the fidelity of the outcomes noted when comparing quality across healthcare facilities.

## 5 Conclusion

In conclusion, this systematic review examined the use of QIs in SACT delivery across international healthcare systems. Existing literature primarily originates from HICs, focusing on breast, colorectal, lung, and skin, the most prevalent tumour groups. The vast majority of QIs evaluated processes of care rather than structural indicators or patient outcomes, the latter due to a lack of integrated data systems. Future avenues of research should focus on understanding how QIs can be developed, implemented, and utilised across differently-resourced healthcare settings, expanding QIs into additional tumour groups, facilitating seamless data collection for appropriate structural and outcome QIs, and integrating PROMs into routine use in cancer care.

## Disclaimers

The views expressed in the submitted article are the authors’ opinions and are not an official position of the institutions.

## Funding

This work was supported by a grant from C/CAN. RS and JT are funded by Medical Research Council Global Alliance for Chronic Diseases Grant ACCI GACD-025.

## CRediT authorship contribution statement

**Kari Leung:** Methodology, Formal analysis, Investigation, Writing – original draft, Visualisation. **Megan McLeod:** Methodology, Formal analysis, Investigation, Writing – original draft, Visualisation. **Julie Torode:** Writing – review and editing. **André Ilbawi:** Writing – review and editing. **Jade Chakowa:** Writing – review and editing. **Brian Bourbeau:** Writing – review and editing. **Manju Sengar:** Writing – review and editing. **Christopher M. Booth:** Writing – review and editing. **Julie R. Gralow:** Writing – review and editing. **Richard Sullivan:** Conceptualization, Methodology, Writing – review and editing, Supervision. **Ajay Aggarwal:** Conceptualization, Methodology, Formal analysis, Investigation, Writing – review and editing, Supervision, Visualisation.

## Declaration of Competing Interest

The authors declare that they have no known competing financial interests or personal relationships that could have appeared to influence the work reported in this paper.
