# Supplementary material for: Quality indicators for systemic anticancer therapy services: a systematic review of metrics used to compare quality across healthcare facilities
Source: Eur J Cancer. 2023 Dec;195:None. doi: 10.1016/j.ejca.2023.113389 (PMC10697827; doi:10.1016/j.ejca.2023.113389)
Supplement: Supplementary file 1 — Supplementary material [file mmc1.docx]

# Appendices

- 1. - Search Strategy

### EMBASE

**Database**: Embase <1974 to 2022 October 31>

| # | Query |
| --- | --- |
| 1 | quality of healthcare.ti. or quality of healthcare.ab. |
| 2 | quality improvement.ti. or quality improvement.ab. |
| 3 | quality indicator.ti. or quality indicator.ab. |
| 4 | quality measure.ti. or quality measure.ab. |
| 5 | performance indicator.ti. or performance indicator.ab. |
| 6 | performance measure.ti. or performance measure.ab. |
| 7 | outcome indicator.ti. or outcome indicator.ab. |
| 8 | outcome measure.ti. or outcome measure.ab. |
| 9 | outcome metric.ti. or outcome metric.ab. |
| 10 | quality metric.ti. or quality metric.ab. |
| 11 | performance metric.ti. or performance metric.ab. |
| 12 | 1 or 2 or 3 or 4 or 5 or 6 or 7 or 8 or 9 or 10 or 11 |
| 13 | cancer.ti. or cancer.ab. |
| 14 | neoplasm.ti. or neoplasm.ab. |
| 15 | tum?r.ti. or tum?r.ab. |
| 16 | oncology.ti. or oncology.ab. |
| 17 | 13 or 14 or 15 or 16 |
| 18 | drug therap*.ti. or drug therap*.ab. |
| 19 | chemotherap*.ti. or chemotherap*.ab. |
| 20 | systemic therap*.ti. or systemic therap*.ab. |
| 21 | exp Antineoplastic Combined Chemotherapy Protocols/ |
| 22 | exp Antineoplastic Protocols/ |
| 23 | 18 or 19 or 20 or 21 or 22 |
| 24 | 12 and 17 and 23 |
| 25 | limit 24 to (human and english language and yr="2000 - 2022" and (article or article in press or books or chapter) and (adult <18 to 64 years> or aged <65+ years>)) |

### PubMed

**Search**: ((quality of healthcare[Title/Abstract]) OR (quality improvement[Title/Abstract]) OR (quality measure[Title/Abstract]) OR (quality metric[Title/Abstract]) OR (performance measure[Title/Abstract]) OR (performance metric[Title/Abstract]) OR (outcome measure[Title/Abstract]) OR (outcome metric[Title/Abstract]) OR (quality indicator[Title/Abstract]) OR (performance indicator[Title/Abstract]) OR (outcome indicator[Title/Abstract])) AND ((chemotherapy[Title/Abstract]) OR (drug therapy[Title/Abstract]) OR (systemic therapy[Title/Abstract])) AND ((cancer[Title/Abstract]) OR (neoplasm[Title/Abstract]) OR ("neoplasms"[mesh]) OR (tumor[Title/Abstract]) OR (tumour[Title/Abstract]) OR (oncology[Title/Abstract])) Filters: English, Adult: 19+ years, Young Adult: 19-24 years, Adult: 19-44 years, Middle Aged + Aged: 45+ years, Middle Aged: 45-64 years, Aged: 65+ years, 80 and over: 80+ years, English, Adult: 19+ years, Young Adult: 19-24 years, Adult: 19-44 years, Middle Aged + Aged: 45+ years, Middle Aged: 45-64 years, Aged: 65+ years, 80 and over: 80+ years, from 2000 – 2022

- 1. - Table of extracted quality indicators from included studies

| Study; year | Tumour group(s); stage | Quality indicators | Donabedian domain | Theme |
| --- | --- | --- | --- | --- |
| Burgers; 2018(31) | Lung; III to IV | Proportion of patients with 30-day mortality after start of systemic treatment for lung cancer | Outcome | Mortality |
| Khorfan; 2021(27) | Lung; I to II | Proportion of patients with pathologic stage II or higher that were recommended chemotherapy, or if not recommended, reason is documented | Process | Appropriateness of care and guideline adherence |
| Van Egmond; 2021(29) | Skin; all stages | Proportion of cases treated with 5-Fluorouracil or imiquimod | Process | Appropriateness of care and guideline adherence |
| O'Neil; 2019(40) | Breast; I to III | Proportion of women age 18-70 years with AJCC stage II-III disease and ER- and PR-negative histology who receive chemotherapy within 120 days from diagnosis | Process | Treatment time intervals |
|  |  | Proportion of women age ≥18 years with AJCC stage I-III disease, tumour size > 1cm, and ER- or PR-positive histology who receive Tamoxifen or an aromatase inhibitor within 365 days from diagnosis | Process | Treatment time intervals |
| Ellis; 2020(28) | Breast, colorectal, lung; all stages | Rates of failure to administer recommended chemotherapy (hospital-level) | Process | Appropriateness of care and guideline adherence |
| Van Bommel; 2017(30) | Breast; all stages | Proportion of patients with standard pathology report including information about ER percentage, PR percentage, HER-2 status, malignancy grade, tumour size, resection margin, and number of positive lymph nodes over those with a pathology report of invasive breast cancer of at least 1cm without neo-adjuvant therapy | Process | Treatment planning and IHC |
|  |  | Proportion of patients with HER-2 positive measurement over those with primary invasive breast cancer and availability of HER-2 status | Process | Treatment planning and IHC |
|  |  | Proportion of patients with ER-positive measurement over those with primary invasive breast cancer and availability of ER status | Process | Treatment planning and IHC |
|  |  | Proportion of patients with PR-positive measurement over those with primary invasive breast cancer and availability of PR status | Process | Treatment planning and IHC |
|  |  | Proportion of patients receiving neo-adjuvant chemotherapy for invasive M0 breast cancer over those with invasive breast cancer treated surgically | Process | Appropriateness of care and guideline adherence |
|  |  | Proportion of patients receiving post-operative chemotherapy for invasive M0 breast cancer over those with invasive breast cancer treated surgically | Process | Appropriateness of care and guideline adherence |
|  |  | Proportion of patients receiving neo-adjuvant or post-operative chemotherapy for invasive M0 breast cancer over those with invasive breast cancer treated surgically | Process | Appropriateness of care and guideline adherence |
|  |  | Proportion of patients receiving neo-adjuvant chemotherapy with transit time ≤5 weeks between diagnosis (date of biopsy) and start of neo-adjuvant chemotherapy | Process | Treatment time intervals |
|  |  | Proportion of patients receiving chemotherapy with transit time ≤5 weeks between final operation and start of chemotherapy | Process | Treatment time intervals |
|  |  | Proportion of patients receiving chemotherapy with transit time ≤5 weeks between end of radiotherapy and start of chemotherapy | Process | Treatment time intervals |
| Mukai; 2015(38) | Breast; all stages | Proportion of patients with breast cancer (excluding unknown ER) with known ER-positive or negative status | Process | Treatment planning and IHC |
|  |  | Proportion of patients with breast cancer who have had HER-2 testing (in cases of HER-2 2+, positive or negative was determined by FISH) | Process | Treatment planning and IHC |
|  |  | Proportion of patients who received post-operative therapy adherent to the St. Gallen consensus recommendation over post-operative cases with invasive breast cancer | Process | Appropriateness of care and guideline adherence |
|  |  | Proportion of patients receiving post-operative hormone therapy (Tamoxifen, Toremifene, anastrozole, exemestane, and letrozole) over post-operative cases with invasive breast cancer positive for ER or PR and tumour diameter of 1cm or larger | Process | Appropriateness of care and guideline adherence |
|  |  | Proportion of patients who received either regimen including anthracyclines, taxanes, or CMF over invasive breast cancer cases with implementation of post-operative adjuvant chemotherapy | Process | Appropriateness of care and guideline adherence |
| Wu; 2021(32) | Breast; I to III | Rates of ED visits and hospitalisations in the 180 days following chemotherapy initiation | Outcome | Adverse events and side effects |
| Inwald; 2019(36) | Breast; I to III | Proportion of patients with early HER-2 positive breast cancer who were recommended Trastuzumab, or if not recommended, reason is documented | Process | Appropriateness of care and guideline adherence |
| Powis; 2017(33) | Breast; I to III | Proportion of patients with at least one consultation with a provider that prescribes chemotherapy within 120 days of surgery | Process | Multidisciplinary and co-ordinated care |
|  |  | Proportion of patients who initiated multi-agent chemotherapy within 120 days of surgery | Process | Appropriateness of care and guideline adherence |
|  |  | Proportion of patients who initiated chemotherapy within 60 days of surgery | Process | Treatment time intervals |
|  |  | Proportion of hormonally-sensitive patients who received adjuvant hormone therapy within 1 year of surgery | Process | Appropriateness of care and guideline adherence |
|  |  | Proportion of patients on hormone therapy with > 80% of eligible days covered with prescription | Process | Multidisciplinary and co-ordinated care |
|  |  | Proportion of patients with at least one billing for chemotherapy within 4 months of surgery with a documented report an ER visit or hospitalisation within 30 days of chemotherapy | Outcome | Adverse events and side effects |
|  |  | Proportion of patients with at least one billing for chemotherapy within 4 months of surgery with a documented report of serious treatment-related neutropenia within 30 days of chemotherapy | Outcome | Adverse events and side effects |
|  |  | Proportion of patients who died within 60 days of adjuvant chemotherapy | Outcome | Mortality |
|  |  | Proportion of patients with at least one prescription filled for GCSF in cycle after ED visit plus hospitalisation episode (2 weeks before to 1 week after) | Process | Supportive medicines |
|  |  | Proportion of patients who filled a prescription for appropriate anti-emetics with their cycle of chemotherapy | Process | Supportive medicines |
|  |  | Proportion of patients eligible for systemic treatment with either ER, PR, or HER-2 status known | Process | Treatment planning and IHC |
|  |  | Proportion of patients with HER-2 negative breast cancer who received trastuzumab within 12 months of diagnosis | Process | Appropriateness of care and guideline adherence |
|  |  | Proportion of patients who received endocrine therapy with ER-positive, PR-negative breast cancer within 1 year of surgery | Process | Appropriateness of care and guideline adherence |
|  |  | Proportion of patients whose first cycle of chemotherapy was ordered using computerised provider order entry system | Structure | Electronic prescribing systems |
| Gray; 2011(26) | Breast; all stages | Proportion of medical charts with documented HER-2 status (determined by IHC or FISH) | Process | Treatment planning and IHC |
|  |  | Proportion of medical charts with documented hormone receptor status both ER and PR (i.e., positive, negative, or percentage documented) | Process | Treatment planning and IHC |
|  |  | Proportion of patients with positive HER-2 status where the physician discussed, recommended or offered treatment with Herceptin | Process | Appropriateness of care and guideline adherence |
|  |  | Proportion of non-metastatic patients with ER-positive or PR-positive breast cancer where the physician discussed, recommended, or referred treatment with Tamoxifen or aromatase inhibitors within one year of diagnosis | Process | Appropriateness of care and guideline adherence |
|  |  | Proportion of stage IV patients with ER-positive or PR-positive breast cancer where the physician discussed, recommended, or referred treatment with Tamoxifen, Fulvestrant, or aromatase inhibitors within one year of diagnosis | Process | Appropriateness of care and guideline adherence |
|  |  | Proportion of patients with stage I, ER-negative or PR-negative disease and tumour size greater than 1 cm where the physician discussed, recommended, or referred for chemotherapy | Process | Appropriateness of care and guideline adherence |
|  |  | Proportion of patients with stage II or III disease where the physician discussed, recommended, or referred for chemotherapy | Process | Appropriateness of care and guideline adherence |
|  |  | Proportion of non-metastatic patients who received adjuvant chemotherapy where the adjuvant chemotherapy regimen was from the provided list | Process | Appropriateness of care and guideline adherence |
|  |  | Proportion of metastatic patients who received chemotherapy where the chemotherapy regimen was from the provided list | Process | Appropriateness of care and guideline adherence |
|  |  | Proportion of patients who received chemotherapy where there was a signed consent for treatment in the chart or practitioner’s note that the treatment was discussed and patient consented to treatment | Process | Treatment planning and IHC |
|  |  | Proportion of patients who received chemotherapy where there was a flowsheet with chemotherapy notes and blood counts | Process | Treatment planning and IHC |
|  |  | Proportion of patients who received chemotherapy where the patient's planned dose of chemotherapy was documented in the medical oncology note | Process | Treatment planning and IHC |
|  |  | Proportion of non-metastatic patients whose planned dose of chemotherapy was documented, where the patient’s planned dose of chemotherapy, dose per cycle, and number of cycles fell within a range that is consistent with published regimens | Process | Appropriateness of care and guideline adherence |
|  |  | Proportion of patients who received chemotherapy where the patient's body-surface area was documented | Process | Treatment planning and IHC |
|  |  | Proportion of non-metastatic patients who received adjuvant chemotherapy where the patient received definitive surgery after neoadjuvant chemotherapy | Process | Multidisciplinary and co-ordinated care |
|  |  | Proportion of non-metastatic patients who received adjuvant chemotherapy where the patient started adjuvant chemotherapy within 8 weeks of completion of surgical therapy | Process | Treatment time intervals |
|  |  | Proportion of non-metastatic patients who received adjuvant chemotherapy where the patient started adjuvant chemotherapy within 4 months of diagnosis | Process | Treatment time intervals |
| Ferreira; 2016(39) | Breast; I | Proportion of patients with stage I breast cancer that received adjuvant chemotherapy | Process | Appropriateness of care and guideline adherence |
| Boyle; 2022(34) | Colorectal; III to IV | Rates of hospital-level severe acute toxicity for patients receiving SACT (i.e., patients requiring an overnight hospital admission with pre-defined ICD-10 diagnostic codes between the administration of the first cycle of SACT and up until 8 weeks after the administration of the last cycle of SACT) | Outcome | Adverse events and side effects |
|  |  |  |  |  |
| Wallington; 2016(35) | Breast, lung; all stages | 30-day mortality rates for breast and lung cancer patients receiving SACT in England | Outcome | Mortality |
| kowalski; 2014(37) | Breast; all stages | Percentage of patients recommended chemotherapy in cases of steroid receptor negative diagnostic finding | Process | Appropriateness of care and guideline adherence |
|  |  | Percentage of patients recommended chemotherapy in cases of receptor positive and nodal positive diagnostic finding | Process | Appropriateness of care and guideline adherence |
|  |  | Percentage of patients recommended endocrine therapy in cases of steroid receptor diagnostic finding | Process | Appropriateness of care and guideline adherence |
|  |  | Percentage of patients recommended Trastuzumab therapy over one year in cases of HER-2-positive diagnostic finding | Process | Appropriateness of care and guideline adherence |
|  |  | Percentage of patients recommended endocrine therapy in cases of metastases | Process | Appropriateness of care and guideline adherence |
|  |  | Percentage of patients with pre-therapeutic histological confirmation | Process | Treatment planning and IHC |

Abbreviations: AJCC, American Joint Committee on Cancer; CMF, Cyclophosphamide, Methotrexate and Fluorouracil; ED, emergency department; ER, oestrogen receptor; FISH, florescence in situ hybridisation; GCSF, granulocyte colony-stimulating factor; HER-2, human epidermal growth factor receptor 2; IHC, immunohistochemistry; PR, progesterone receptor; SACT, systemic anti-cancer therapy
